# Genetic and Biochemical Characterization of an Exopolysaccharide With *in vitro* Antitumoral Activity Produced by *Lactobacillus fermentum* YL-11

**DOI:** 10.3389/fmicb.2019.02898

**Published:** 2019-12-17

**Authors:** Yunlu Wei, Fei Li, Le Li, Linlin Huang, Quanhong Li

**Affiliations:** ^1^College of Food Science and Nutritional Engineering, China Agricultural University, Beijing, China; ^2^National Engineering Research Center for Fruit and Vegetable Processing, Beijing, China; ^3^Department of Environmental and Quality Inspection, Chongqing Chemical Industry Vocational College, Chongqing, China

**Keywords:** *Lactobacillus fermentum*, exopolysaccharide, whole genome sequence, biosynthetic mechanism, antitumoral activity

## Abstract

In the present study, the whole genome sequence of *Lactobacillus fermentum* YL-11, a novel exopolysaccharide (EPS)-producing lactic acid bacteria (LAB) strain isolated from fermented milk, was determined. Genetic information and the synthetic mechanism of the EPS in *L. fermentum* YL-11 were identified based on bioinformatic analysis of the complete genome. The purified EPS of YL-11 mainly comprised galactose (48.0%), glucose (30.3%), mannose (11.8%), and arabinose (6.0%). *In vitro*, the EPS from YL-11 exhibited inhibition activity against HT-29 and Caco-2 colon cancer cells, suggesting that EPS from strain YL-11 might be used as an antitumoral agent. EPS at 600 and 800 μg/mL achieved inhibition rates of 46.5 ± 3.5% and 45.6 ± 6.1% to HT-29 cells, respectively. The genomic information about *L. fermentum* YL-11 and the antitumoral activity of YL-11 EPS provide a theoretical foundation for the future application of EPS in the food and pharmaceutical industries.

## Introduction

Lactic acid bacteria (LAB) have received generally recognized as safe (GRAS) status due to their long history of safe use in a wide range of fermented food products. In addition, LAB-derived metabolites, such as organic acids, bacteriocins, vitamins, low-calorie polyols, exopolysaccharides (EPSs), and hyaluronic acid, play important roles in the food and pharmaceutical industries (Gaspar et al., 2013).

EPSs secreted by LAB have been widely studied because of their potential beneficial effects for human health. Specifically, their application in improving the physical properties of fermented food has attracted attention (Yildiz and Karatas, 2018). Bacterial EPSs are high molecular weight carbohydrate polymers, composed of monosaccharides (units) linked together by glycosylic linkages, which are closely attached to the bacterial surface or are released into the surrounding environment (Zeidan et al., 2017). EPSs produced by LAB have been reported to exhibit antioxidant (Sivasankar et al., 2018), antitumoral (Rajoka et al., 2018), and immunological activities (Mitsuoka, 1992; Nagai et al., 2011); cholesterol-lowering and antibiofilm properties (Mahdhi et al., 2017); and can improve the intestinal flora balance (Castro-Bravo et al., 2018). Among these health benefits, the antitumoral activity of EPSs has attracted increasing attention because of the enhanced number and incidence of cancer.

Some studies on the antitumoral activities of EPSs from LAB strains (e.g., *Lactobacillus acidophilus*, *Lactobacillus plantarum*, *Lactobacillus rhamnosus*, and *Lactobacillus casei*, etc.) have been carried out. It was suggested that EPS from *L. acidophilus* 10307 could induce cytotoxicity in two colon cancer cell lines, HCT15 and Caco2 cells (Deepak et al., 2016). Additionally, EPS from *L. plantarum* WLPL04 and *L. plantarum* YW32 showed significant inhibitory effects on HT-29 colon cancer cells (Wang et al., 2015b; Liu et al., 2017); EPS from *L. plantarum* RJF_4_ displayed antitumoral activity in pancreatic cancer cells (Dilna et al., 2015). cell-bound EPS from *L. rhamnosus* ATCC 9595 could inhibit the growth of PANC1 and HT-29 cancer cells (Kim et al., 2006). *In vitro* antitumor analysis demonstrated that the acidic EPS of *L. casei* SB27 had an anti-proliferative effect on HT-29 cells (Di et al., 2017). These results suggested basis that EPSs from LAB might be safe natural antitumor agents.

Many EPS-producing LAB species have been isolated and identified, including *Streptococcus thermophilus* (Lemoine et al., 1997), *Lactococcus lactis* (Liu et al., 2013), *L. casei*, *L. rhamnosus*, *L. plantarum*, *Lactobacillus delbrueckii* subsp. *bulgaricus*, *Lactobacillus brevis*, *Lactobacillus curvatus*, *Lactobacillus helveticus*, *Lactobacillus johnsonii* (Oleksy and Klewicka, 2018), and *Lactobacillus fermentum* (Ale et al., 2016). Among these LAB species, *L. fermentum* has attracted attention because it is often used as a starter culture in fermentation and nutritional supplements for complementary therapies (López-Huertas, 2014). The *L. fermentum* CECT5716 strain, originally isolated from human breast milk, was the first commercial *L. fermentum* strain in dietary supplement products. Research has shown that CECT5716 strain exhibited potential mastitis prevention, immunity support, and prevention of infant colic activities (Maldonado-Lobón et al., 2015). The EPS from *L. fermentum* TDS030603 displayed stronger viscosity than xanthan gum (Dan et al., 2009). However, the biosynthetic mechanism and probiotic application of *L. fermentum* EPS remains to be explored.

In the present study, a novel EPS-producing LAB strain isolated from fermented milk was identified as *L. fermentum* YL-11. The complete genome of this strain YL-11 was determined to further analyze the EPS biosynthetic mechanism.

## Materials and Methods

### Bacterial Strains and Culture Conditions

A total of 155 LAB strains were isolated from five Chinese traditional yogurts samples from which the milk was derived from cows. They were selected from De Man, Rogosa, and Sharpe (MRS) agar plates by colony identification and Gram staining. These strains were stored at −80°C in MRS broth with 20% (v/v) glycerol. EPS fermentation medium with 10% (w/v) reconstituted skim milk was prepared according to the method of Torino (Torino et al., 2001).

### Screening of EPS-Producing LAB

The screening of EPS-producing LAB was performed on MRS agar plates. The previously isolated and stored strains were streaked on MRS agar plates and incubated at 37°C for 48 h (Smitinont et al., 1999; Feng et al., 2012). The isolated strains that displayed mucoid (or ropy) phenotypes were recorded as capable of producing EPS. To determine the yield of EPS production, each isolate was inoculated into EPS fermentation medium at 37°C for 24 h and the EPS was then extracted from the culture medium using ethanol precipitation and their concentration was measured using the phenol sulfuric acid method (Cuesta et al., 2003).

### Identification of EPS-Producing LAB Strain, *L. fermentum* YL-11

The genomic DNA of YL-11 strain was extracted and purified using a QIAamp DNA Mini Kit (Qiagen, Hilden, Germany). The DNA quality was evaluated using a NanoDrop 2500 spectrophotometer (Thermo Fisher Scientific, Waltham, MA, United States). The species level of theYL-11 strain was identified using 16S rDNA sequencing analysis. Amplification of the 16S rDNA sequence was performed according to a previously published method (Li et al., 2012), and the primers sequences were as follows: 27F: 5′-AGAGTTTGATCCTGGCTCAG-3′ and 1492R: 5′-GGTTACCTTGTTACGACTT-3′. The amplified 16S rDNA products were then purified, sequenced, and compared with sequences deposited in the GenBank database.

### Genome Sequencing and Assembly

The complete genome of strain YL-11 was sequenced on the Illumina Hiseq 2000 platform (2 × 100 bp). Gap closing was performed using PCR and Sanger sequencing. The genome sequences were assembled by SOAPdenovo^1^.

### Genome Gene Annotation

The whole genome of strain YL-11 was annotated via Rapid Annotation Subsystem Technology (RAST). Identification of coding proteins was predicted by using databases of the Kyoto Encyclopedia of Genes and Genomes (KEGG) and the Cluster of Orthologous Groups of proteins (COG) databases (Valeriano et al., 2019). The EPS genes were aligned using the NCBI database.

### Isolation and Purification of EPS

The EPS fermentation medium was inoculated with 5% (v/v) overnight YL-11 culture suspensions and incubated at 37°C for 48 h, under static conditions. The EPS was extracted from the culture medium using the protocol described by Goh, with some modifications (Goh et al., 2005). In brief, at the end of the fermentation, the fermented milk was heated at 100°C for 10 min to inactivate the enzymes. The supernatant was collected by centrifugation at 8000 × *g* for 15 min at 4°C and then adjusted to pH 7.5 using 1 M NaOH. The crude EPS extract was then subjected to hydrolysis, ethanol precipitation, dialysis, and lyophilization. The EPS concentration was measured using the phenol sulfuric acid method (Cuesta et al., 2003).

The EPS was further purified using column chromatography. The lyophilized crude EPS was fractionated with a DEAE Fast Flow anion-exchange chromatography column (GE, Sweden) with a gradient of 0.1, 0.2, 0.3, 0.4, and 0.5 M NaCl at a flow rate of 1 mL/min. The fractions were collected and analyzed using the phenol sulfuric acid method (Cuesta et al., 2003). The fractions collected from the anion-exchange chromatography column were further purified by a Sephadex CL-6B column (1.6 cm × 60 cm, GE, Sweden) with 0.005 M NaCl at a flow rate of 0.5 mL/min. Finally, the purified fractions were dialyzed and lyophilized.

A high performance gel permeation chromatography (HPGPC) system equipped with a Shodex SB-805M HQ column (8.0 × 300 mm, Showa Denko KK, Miniato, Japan) and a refractive index (RI) detector was used to analyze the lyophilized EPS. The 0.1 M NaNO_3_ at a flow rate of 0.8 mL/min was employed as elution buffer.

### Monosaccharide Composition Analysis

The monosaccharide composition of EPS was analyzed by gas chromatography-mass spectrometry (GC-MS) (Shimadzu QP 2010 plus, Japan) with a flame ionization detector (FID) and an Rxi-5 Sil MS column (30 m × 0.25 mm × 0.25 μm). Briefly, 2 mg of EPS samples were hydrolyzed with 1 mL of 2 M trifluoroacetic acid (TFA) for 90 min, after which the acid was removed by evaporation. The hydrolysate was washed using milliQ water and dried. The dried hydrolysate was then reduced with sodium borohydride and acetylated using acetic anhydride (Du et al., 2018). The derivative was subjected to GC-MS analysis. Standards of rhamnose, halidose, arabinose, xylose, mannose, galactose, and glucose were prepared for composition analysis.

### Scanning Electron Microscopy

The purified EPS samples were fixed to conductive tape and coated with a gold layer at about 10 nm thick. After processing, samples were observed using a scanning electron microscope (FEI, Quanta 200, Thermo Fisher Scientific) at an acceleration voltage of 20 KV (Prasanna et al., 2012).

### Antitumoral Activity of EPS

The antitumoral activity of YL-11 EPS was assessed using a Cell Counting Kit-8 (CCK-8, Med Chem Express, Monmouth Junction, NJ, United States). Colon cancer cells (HT-29 and Caco-2 cells) were cultured in Dulbecco’s modified Eagle’s medium (DMEM) with 10% fetal bovine serum (FBS) and 1% Penicillin-Streptomycin in a humidified incubator at 37°C, 5% CO_2_. Normal colon cells (NCM460 cells) were cultured in Roswell Park Memorial Institute (RPMI) 1640 medium with 10% FBS and 1% Penicillin-Streptomycin in a humidified incubator at 37°C, 5% CO_2_. The cells suspensions were obtained by digestion with 0.25% trypsin and then inoculated at 100 μl/well into 96-well plates. After incubating the plates at 37°C for 24 h, different concentrations of EPS (0, 50, 100, 200, 400, 600, and 800 μg/mL), and 50 μg/mL 5-Fluorouracil (5-Fu) as positive control for colon cancer cell lines (HT-29 and Caco-2), were added to the cells for 12, 24, and 48 h (Rajoka et al., 2018). Medium from each well was renewed with 100 μL of fresh medium without drugs, and 10 μL CCK-8 solution was then added. The cells were incubated at 37°C for 1 h. The inhibition of cell proliferation was measured via the absorbance at 450 nm using a microplate reader (Spark10M, Tecan, Switzerland).

Hoechst staining was performed according to the manufacturer’s protocol (Yaoxian et al., 2013). HT-29 cells at 5 × 10^5^ cells/mL were incubated in 24-well plates using a CO_2_ incubator at 37°C for 24 h. The cells were then treated with different concentrations of EPS for 48 h. The cells treated were washed twice with phosphate-buffered saline (PBS) and fixed with 4% paraformaldehyde at room temperature for 20 min. After fixation, the cells were washed twice with PBS and incubated with Hoechst 33342 dye (Solarbio, Beijing, China) for 5 min at room temperature in the dark. Then, the dye solutions were removed and the stained cells were washed twice with PBS, and then viewed and photographed under an inverted fluorescence microscope (Axio Observer A1, Zeiss AG, Oberkochen, Germany).

### Statistical Analysis

All experiments were conducted in triplicate. Results were analyzed by one-way analysis of variance (ANOVA). All results are presented as the mean ± standard deviations (SD) and a *P*-value < 0.05 was considered statistically significant.

## Results

### Isolation of EPS-Producing Strains and Identification of Strain YL-11

Among these 155 strains isolated from Chinese traditional yogurts samples, 11 LAB isolates that displayed mucoid (or ropy) phenotypes were recorded as capable of producing EPS. One strain, YL-11, was observed to produce EPS at a maximum yield of 84.5 mg/L in skim milk medium, as shown in Supplementary Table S1. Strain YL-11 was then identified to be of the species of *L. fermentum* by 16S rDNA sequencing analysis.

### Genome Features of *L. fermentum* YL-11

**FIGURE 1:**
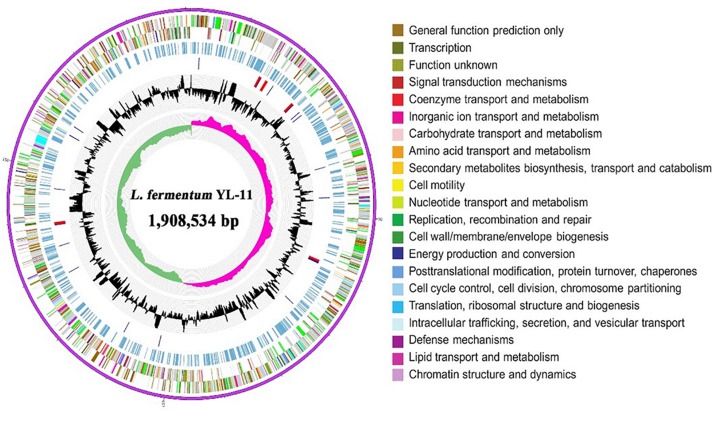
Circular genomic map of *L. fermentum* YL-11. The first ring represents the genome sequence, the 2nd and 3rd rings represent the predicted COG annotated coding sequences, the 4th ring shows the KEGG-related enzymes, the 5th and 6th rings indicate RNA genes and the GC content, respectively, and the innermost ring (7) shows the GC skew. Very short features were enlarged to enhance visibility. Clustered genes, such as several rRNA genes, may appear as one-line due to space limitations.

The complete genome of *L. fermentum* YL-11 was determined, and comprised a single, circular chromosome of 1,908,534 bp (Figure 1). The total number of reads and contigs after assembly were 4308714 and 56, respectively. The average GC content of the chromosome was 51.9% and there were 1843 total predicted CDS, 58 tRNA genes and 15 rRNA genes. The Cluster of Orthologous Groups (COG) analysis of the encoded proteins from the *L. fermentum* YL-11 genome revealed the genetic basis for its characteristics (Table 1). The genome information was deposited in the GenBank database under the accession no. CP034193.

**TABLE 1:** Cluster of orthologous groups categories of *L. fermentum* YL-11.

**COG class**	**Name**	**Count**	**Proportion**
C	Energy production and conversion	77	4.12%
D	Cell cycle control, cell division, chromosome partitioning partitioning	29	1.55%
E	Amino acid transport and metabolism	178	9.51%
F	Nucleotide transport and metabolism	97	5.18%
G	Carbohydrate transport and metabolism	126	6.73%
H	Coenzyme transport and metabolism	99	5.29%
I	Lipid transport and metabolism	77	4.12%
J	Translation, ribosomal structure and biogenesis	186	9.94%
K	Transcription	107	5.72%
L	Replication, recombination and repair	96	5.13%
M	Cell wall/membrane/envelope biogenesis	105	5.61%
N	Cell motility	12	0.64%
O	Posttranslational modification, protein turnover, chaperones	54	2.89%
P	Inorganic ion transport and metabolism	98	5.24%
Q	Secondary metabolites biosynthesis, transport and catabolism	25	1.34%
R	General function prediction only	173	9.25%
S	Function unknown	90	4.81%
T	Signal transduction mechanisms	67	3.58%
U	Intracellular trafficking, secretion, and vesicular transport	14	0.75%
V	Defense mechanisms	44	2.35%
W	Extracellular structures	3	0.16%
X	Mobilome: prophages, transposons	114	6.09%

### Exopolysaccharide Biosynthesis Analysis

**FIGURE 2:**
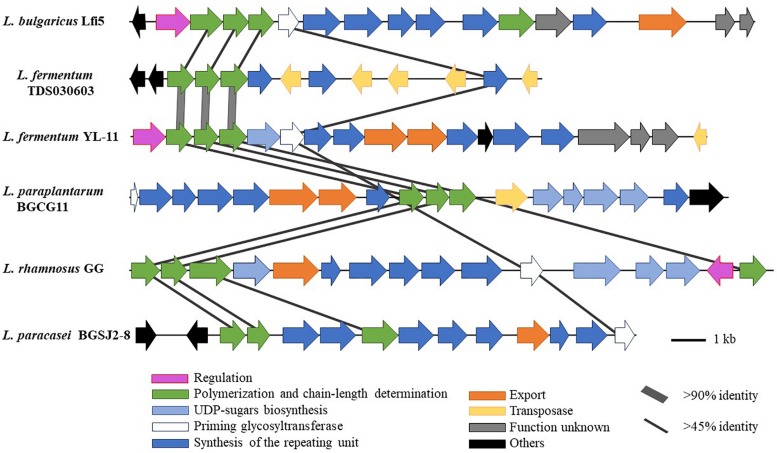
Genetic graph of EPS biosynthesis gene clusters of *L. fermentum* YL-11 and other *Lactobacillus* strains. The EPS gene clusters in *L. fermentum* YL-11 (CP019348), *L. bulgaricus* Lfi5 (AF267127), *L. fermentum* TDS030603 (AB519644), *L. paraplantarum* BGCG11 (HG316787), *L. rhamnosus* GG (FJ428614) and *L. paracasei* BGSJ2-8 (LN879393) are showed by arrows with different sizes and colors.

**FIGURE 3:**
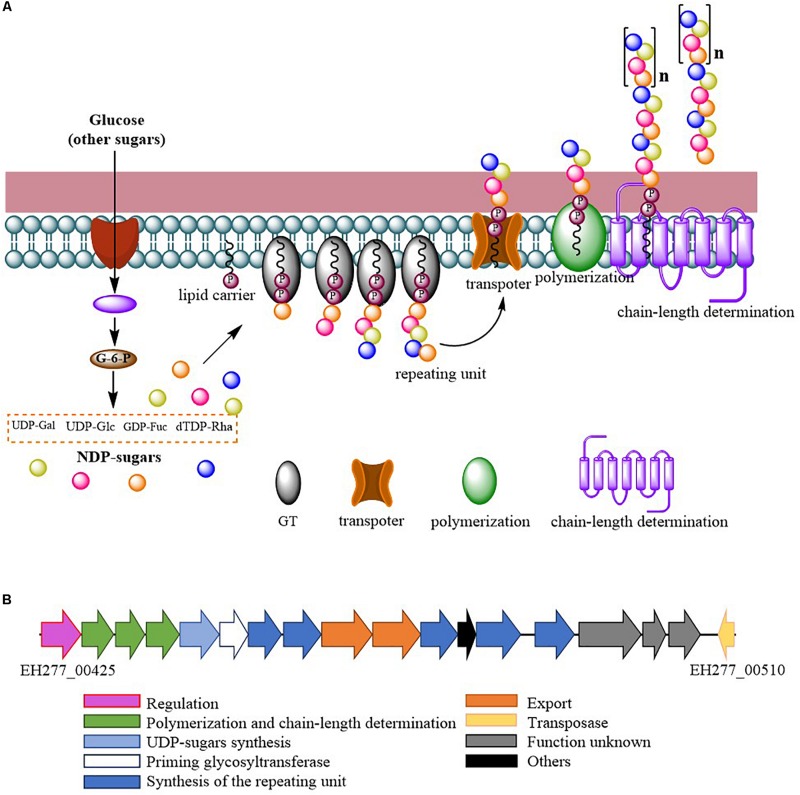
**(A)** Proposed biosynthetic model of exopolysaccharide production by *L. fermentum* YL-11; **(B)** Genetic organization of the EPS gene cluster in *L. fermentum* YL-11.

Comparing the EPS encoding sequences of LAB, the partial EPS gene cluster about 16.6 kb in the genome of *L. fermentum* YL-11 was determined. Within the EPS gene cluster of strain YL-11, 18 putative open reading frames (ORFs), including some of unknown function, were identified according to computational analysis (Supplementary Table S2). Generally, the biosynthetic process of EPSs in LAB comprises four types of functional proteins: regulation; biosynthesis of repeating units; polymerization and chain-length determination; and export (Lamothe et al., 2002). A comparison between the genetic organization of EPS gene clusters in *L. fermentum* YL-11 (CP019348), *L. bulgaricus* Lfi5 (Lamothe et al., 2002), *L. fermentum* TDS030603 (Dan et al., 2009), *L. paraplantarum* BGCG11 (Zivkovic et al., 2015), *L. rhamnosus* GG (Lebeer et al., 2009), and *L. paracasei* BGSJ2-8 (Živković et al., 2016) is provided in Figure 2. A comparison with the EPS gene clusters in these other *Lactobacillus* strains suggested that the EPS gene cluster in *L. fermentum* YL-11 also contained genes encoding the four types of functional proteins. According to the bioinformatic analysis, a biosynthetic model of EPS in *L. fermentum* YL-11 was proposed (Figure 3A). As shown in Figure 3B, the biosynthetic process was executed using the following proteins. A LytR family transcriptional regulator protein (EH277_00425), which is often responsible for the regulation of EPS synthesis, was identified. Three proteins (EH277_00430, EH277_00435, and EH277_00440) were involved in the polymerization and chain length determination process. The gene (EH277_00430), located downstream of the regulatory gene (EH277_00425), encodes a protein that is a homolog of a polysaccharide biosynthesis tyrosine autokinase in *L. fermentum* TDS030603 (LF25067_RS00535). The precursor gene (EH277_00445)

encodes an NAD-dependent epimerase/dehydratase family protein, which could be involved in the biosynthesis of EPS precursors (Boels et al., 2001). Six genes (EH277_00450, EH277_00455, EH277_00460, EH277_00475, EH277_00485, and EH277_00490) were identified to encode putative glycosyltransferases, which are involved in the biosynthesis of the repeating units. The export gene products contain a protein (EH277_00465) that is similar to the membrane protein of *L. reuteri* ATCC 55730 (lr2135, 34% identity) and another encodes a putative capsular polysaccharide synthesis protein (EH277_00470), which might also be involved in the export of EPS. The gene (EH277_00510) encoding a transposase which is homologous to the transposase of *L. fermentum* 3872 (N573_000575, 85% identity) was identified.

### Purification and Monosaccharide Compositions of YL-11 EPS

**FIGURE 4:**
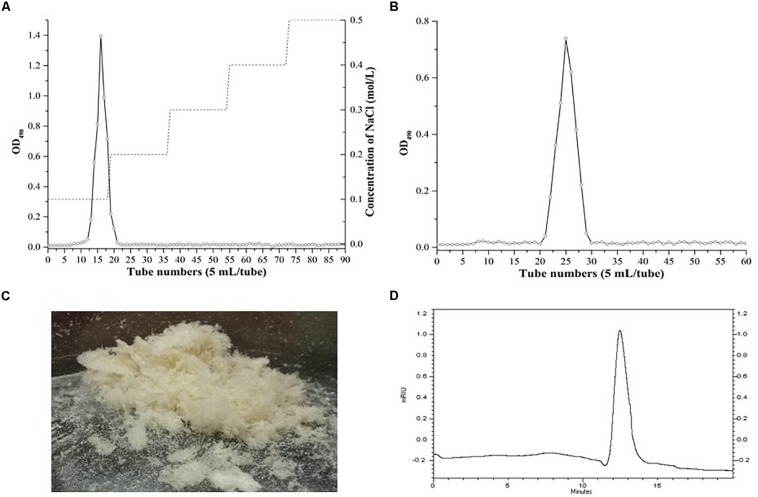
Purification of the EPS produced by *L. fermentum* YL-11. **(A)** DEAE-FF anion exchange chromatogram. **(B)** Sephadex CL-6B gel filtration chromatogram. **(C)** The aspect of EPS. **(D)** HPGPC (high performance gel permeation chromatography) of EPS.

**FIGURE 5:**
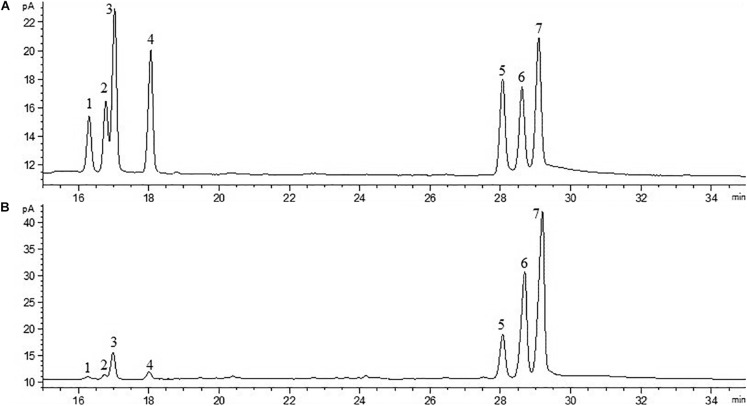
Chromatogram of standard monosaccharide **(A)** and EPS monosaccharide compositions **(B)** (1-rhamnose; 2-halidose; 3-arabinose; 4-xylose; 5-mannose; 6-glucose; and 7-galactose).

The crude EPS (84.5 ± 2.5 mg/L) extracted from medium was further purified (Figure 4). After the EPS was fractionated using a DEAE anion exchange column, only one peak was eluted with 0.1 M NaCl (Figure 4A). The corresponding fraction comprising 24.3 ± 1.8 mg of EPS was then loaded onto a Sephadex CL-6B gel filtration column. As shown in Figure 4B, a single peak was eluted, collected, dialyzed, and lyophilized for further analysis. The lyophilized fraction from the gel filtration column was a white powder (Figure 4C) and determined to contain 12.7 ± 1.1 mg EPS. Analysis using HPGPC indicated that the purified lyophilized EPS formed the only symmetrical peak (Figure 4D), revealing the high quality of the purified EPS. The results of monosaccharide composition analysis revealed that the EPS from strain YL-11 mainly comprised galactose (48.0%), glucose (30.3%), mannose (11.8%), and arabinose (6.0%) (Figure 5).

### Scanning Electron Microscopy Analysis of YL-11 EPS

**FIGURE 6:**
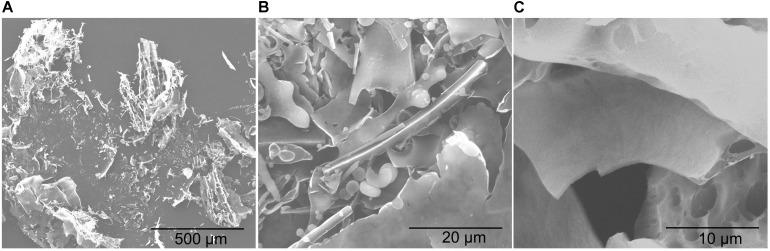
Scanning electron micrograph of *L. fermentum* YL-11 EPS. **(A)** 100×; **(B)** 2000×; **(C)** 5000×.

The SEM analysis showed that YL-11 EPS was lightly attached to the conductive tape, with a slide surface morphology and a homogeneous sheet-like structure (Figure 6). Ahmed et al. (2013) reported another ZW3 EPS produced by *Lactobacillus kefiranofaciens* ZW3 with smooth surface and homogeneous matrix structure, which was similar to YL-11 EPS with aspect to its SEM morphology, and revealed it had good properties that can be used for plasticized film formation. Thus, these surface microstructures of YL-11 EPS might benefit for its use as plasticized film. The surface microstructure of *L. fermentum* YL-11 EPS was also similar to that of the EPS produced by *Leuconostoc citreum* N21 (Yang et al., 2019) and the LW1 and LW2 EPS produced by *L. casei* SB27 (Di et al., 2017).

### Antitumoral Activity of YL-11 EPS

**FIGURE 7:**
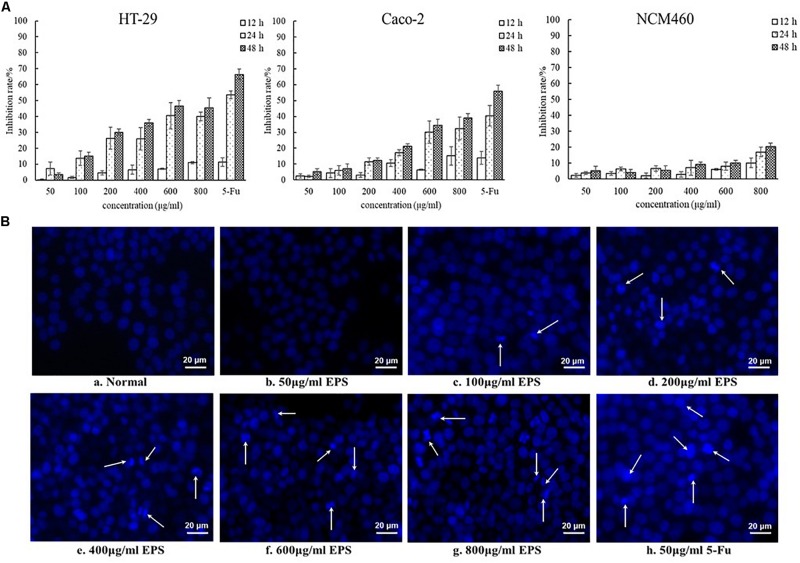
The antitumoral activity of YL-11 EPS against colon cancer cells (HT-29 and Caco-2 cells) and normal colon cells (NCM 460 cells). **(A)** The inhibition rate of EPS on HT-29, Caco-2, and NCM460 cells. All values are expressed as mean ± SD of three independent analyses. **(B)** Results from the Hoechst staining of HT-29 cells. a: normal control; b, c, d, e, f, g, and h: treated with different concentrations (50, 100, 200, 400, 600, and 800 μg/mL, respectively), of EPS and 50 μg/mL 5-Fu.

The EPS-induced inhibition of the growth of colon cancer HT-29 and Caco-2 cells was assessed using different concentrations of EPS (ranging from 50 to 800 μg/mL) at 12, 24, and 48 h (Figure 7A). The concentration-dependent effects of EPS on HT-29 and Caco-2 cell growth were observed. The antitumor effect of EPS on HT-29 cells was stronger than that on Caco-2 cells. After treatment for 48 h, EPS at 600 and 800 μg/ml exhibited no-significant difference in growth inhibition of HT-29 cells, with inhibition rates of 46.5 ± 3.5 and 45.6 ± 6.1%, respectively. To assess the cytotoxicity of EPS toward normal cells, the inhibition rate of NCM460 cells (a normal human colon cell line) treated with EPS was detected. Concentrations of EPS of 50–600 μg/mL showed low toxicity toward normal cells, with an inhibition rate below 10% at 48 h. Hoechst staining of nuclei was then performed to investigate whether the antitumor effects of the EPS on HT-29 cells was associated with the induction of apoptosis. Apoptosis is a physiological process of cell death, which is characterized by morphological changes and nuclear fragmentation (Yang et al., 2013). As shown in Figure 7B, the normal cells without EPS treatment had round regular nuclei, which were considered as normal morphology. However, cells treated with different concentrations EPS and 5-Fu (as a positive control) showed strong blue fluorescence and nuclear condensation consistent with apoptosis. The number of apoptotic cells (white arrows) among cells treated with EPS increased in a concentration-dependent manner, which indicated that EPS-induced apoptosis might inhibit cell proliferation.

## Discussion

Exopolysaccharide have been extracted from other strains of *L. fermentum*, for example, the EPS from *L. fermentum* TDS030603 (Gerwig et al., 2013) and *L. fermentum* Lf2 (Ale et al., 2016) have been characterized. However, it is necessary to investigate the mechanism of EPS biosynthesis by combining the genomic data. Although the genomic information related to EPS biosynthesis has been found in a few strains, including *L. fermentum* F-6 (Sun et al., 2015), *L. fermentum* 222 (Illeghems et al., 2015), *L. fermentum* 3872 (Lehri et al., 2017), and *L. fermentum* MTCC 25067 (Formerly TDS030603) (Aryantini et al., 2017), the mechanism of EPS biosynthesis requires detailed study. In the present study, the gene features and mechanism of EPS biosynthesis in the novel strain *L. fermentum* YL-11 were determined, which might provide the basis for further metabolic engineering and applications of EPS.

According to homology alignment of EPS genes, 13 ORFs in the EPS cluster of *L. fermentum* YL-11 were determined to be related to the EPS biosynthesis process. The predicted products of polymerization and chain length determination genes (EH277_00430, EH277_00435, and EH277_00440) were homologous to the regulatory membrane proteins involved in EPS production (Zeidan et al., 2017). The amino sequences of the proteins encoded by these three genes in *L. fermentum* YL-11 are highly similar to those in *L. fermentum* TDS030603, which were considered to be involved in the polymerization and chain-length determination process of EPS (Dan et al., 2009). The biosynthesis of the EPS repeating unit also requires several glycosyltransferases (GTs), and the encoding genes are often present in the middle part of the EPS gene cluster (Schmid et al., 2015). The precursor gene (EH277_00445) and six putative GTs genes (EH277_00450, EH277_00455, EH277_00460, EH277_00470, EH277_00485, and EH277_00490) in EPS cluster of strain YL-11 were determined. The predicted product of the precursor gene (EH277_00445) is similar to the UDP-glucose 4-epimerase protein of *L. fermentum* NCC2970 (LACFE_CDS0975). The UDP-glucose 4-epimerase could regulate the biosynthesis of precursors in EPS production from *Bifidobacterium longum* subsp. *longum* CRC 002 (Audy et al., 2010). Although the predicted functions of the GT genes in strain YL-11 were the same glycosyltransferases, the nucleotide sequences of these genes are different from those in other *Lactobacillus* species to some extent. Therefore, the activities of these glycosyltransferases might vary, and could provide an opportunity to improve the EPS yield by genetic modification.

The extraction and purification of EPS from the culture medium are essential steps to analyze its physical, chemical, and biological features. Ion exchange and gel filtration chromatography are commonly used to obtain a high purity EPS (Prasanna et al., 2012; Zhu et al., 2018). In the current study, the purified YL-11 EPS obtained from chromatography was subjected to HPGPC analysis. The results suggested that this EPS comprised pure polysaccharides and provided the basis for further study of its structure and bioactivities. Many analytical strategies, e.g., GC-MS, Nuclear magnetic resonance (NMR), Fourier transform infrared (FTIR) spectroscopy, and atomic force microscopy, have been previously applied for the structure determination of EPS. The GC-MS technique has been used to analyze the monosaccharide composition and ratio of EPS (Ricciardi et al., 2002). Similarly, GC-MS analysis showed that glucose and galactose were the main components of EPS from *Bifidobacterium longum* W11 (Inturri et al., 2017). In the present study, we found that the EPS from strain YL-11 mainly comprised galactose (48.0%), glucose (30.3%), mannose (11.8%), and arabinose (6.0%). According to Das et al., analysis using ^1^HNMR and ^13^CNMR spectroscopy revealed that the glucan produced by *L. plantarum* DM5 was composed of 86.5% of α-(1→6) and 13.5% of α-(1→3) linkages (Das and Goyal, 2014). The functional groups of purified EPS from *L. plantarum* YW11 isolated from Tibet Kefir were determined by FTIR spectroscopy (Wang et al., 2015a).

The antitumoral activities of some EPSs from LAB strains have been studied previously. cell-bound EPS from *L. plantarum* 70810 showed significant antitumoral activities against HepG-2, BGC-823, especially HT-29 cancer cells (Wang et al., 2014). LHEPS-2 and LHEPS-3 from *L. helveticus* MB2-1 could inhibit the growth of BGC-823 gastric cancer cells (Li et al., 2014) and Caco-2 human colon cancer cells (Li et al., 2015). The acidic EPS from some *Lactobacillus* strains (such as strain *L. casei* SB27) also showed anti-proliferative activity to some extent (Di et al., 2017). To understand the potential antitumoral activity of YL-11 EPS, the colon tumor cell lines HT-29 and Caco-2 were treated with various concentrations of EPS *in vitro*. After 48h treatment, the EPS from strain YL-11 showed a marked antitumor effect against the HT-29 and Caco-2 cells. The concentrations of 600 and 800 μg/mL YL-11 EPS achieved inhibition rates of 46.5 ± 3.5% and 45.6 ± 6.1% to HT-29 cells, respectively. Thus, the EPS extracted from *L. fermentum* YL-11 has the potential to be applied as an antitumoral agent.

In addition, the EPS from strain YL-11 induced apoptosis of HT-29 cells to some extent. This phenomenon was consistent with the characteristics of other bacterial EPSs with antitumoral activities. The EPS isolated from *L. acidophilus* 606 caused the early death of cancer cells, partly through the induction of apoptosis (Choi et al., 2006). It was reported that the EPS from *L. plantarum* NCU116 induced apoptosis of CT26 cells via TLR2 and Fas/Fasl signaling pathways (Zhou et al., 2017). Di et al. (2018) found that the acidic EPS produced by *L. casei* SB27 could significantly inhibit the growth cancer cells via caspase-3-dependent apoptosis. Thus, the antitumoral mechanism of the EPS from *L. fermentum* YL-11 against colon cancer cells might be induced by apoptosis, but it requires further study. The further investigations on the antitumoral mechanism of YL-11 EPS are in progress by using transmission electron microscope, laser scanning confocal microscopy, flow cytometry, western blot, real-time PCR, and enzyme-linked immunosorbent assays. Additionally, *in vivo* assays of the antitumoral activity and its mechanisms should also be carried out to further understand the detailed functions of the EPS from *L. fermentum* YL-11.

## Conclusion

In conclusion, this study characterized an EPS from a novel strain *L. fermentum* YL-11. The biosynthetic model and gene cluster of YL-11 EPS were determined though bioinformatic analysis. EPS from strain YL-11 was mainly composed of galactose (49.2%), glucose (31.1%), mannose (12.1%), and arabinose (6.1%), making it similar to other EPSs from LAB. The YL-11 EPS exhibited an *in vitro* antitumor effect toward colon cancer cells but not on normal cells. Thus, YL-11 EPS might have potential applications in the food and pharmaceutical industries. In future studies, the structure of YL-11 EPS and its antitumoral mechanism should be investigated.

## Data Availability Statement

The datasets generated for this study can be found in NCBI databases, CP034193.

## Author Contributions

YW and QL designed the experiments and prepared the manuscript. YW, FL, LL, and LH performed the experiments and data analysis with the help of all authors.

## Conflict of Interest

The authors declare that the research was conducted in the absence of any commercial or financial relationships that could be construed as a potential conflict of interest.

## Footnotes

1 http://soapdenovo2.sourceforge.net/
