# Supplementary material for: Genetic and Biochemical Characterization of an Exopolysaccharide With in vitro Antitumoral Activity Produced by Lactobacillus fermentum YL-11
Source: Front Microbiol. 2019 Dec 17;10:2898. doi: 10.3389/fmicb.2019.02898 (PMC6929415; doi:10.3389/fmicb.2019.02898)
Supplement: Supplementary file 1 [file Table_1.docx]

Table S1 EPS production of eleven lactic acid bacterial strains

| Strains | EPS (mg/L) | Identification |
| --- | --- | --- |
| M-28 | 20.3±1.5 | *Lactobacillus acidophilus* |
| M-35 | 25.8±0.8 | *Lactobacillus plantarum* |
| JL-8 | 51.6±5.3 | *Lactococcus lactis* |
| JL-9 | 19.5±2.5 | *Lactobacillus plantarum* |
| ML-6 | 71.7±2.8 | *Lactobacillus plantarum* |
| ML-12 | 43.7±2.3 | *Streptococcus thermophilus* |
| ML-33 | 64.7±1.9 | *Lactobacillus fermentum* |
| YL-11 | 84.5±2.5 | *Lactobacillus fermentum* |
| YL-25 | 31.2±2.4 | *Lactobacillus casei* |
| YL-29 | 52.8±2.3 | *Lactobacillus casei* |
| ZL-15 | 46.7±5.9 | *Lactococcus lactis* |
