# Supplementary material for: Genetic and Biochemical Characterization of an Exopolysaccharide With in vitro Antitumoral Activity Produced by Lactobacillus fermentum YL-11
Source: Front Microbiol. 2019 Dec 17;10:2898. doi: 10.3389/fmicb.2019.02898 (PMC6929415; doi:10.3389/fmicb.2019.02898)
Supplement: Supplementary file 2 [file Table_2.docx]

Table S2 Gene organizations of EPS biosynthesis gene clusters of *L. fermentum* YL-11

| Open reading frame  (Size aa) | Predicted function | Best BLAST match  product | Source organism | Accession No. | Amino acid  identity (%) |
| --- | --- | --- | --- | --- | --- |
| EH277_00425 (335) | Regulation of EPS synthesis | LytR family transcriptional regulator protein | *Lactobacillus fermentum* | TFZ18200.1 | 97 |
| EH277_00430 (256) | Polymerization and chain-length determination | exopolysaccharide biosynthesis protein  Wzz | *Lactobacillus fermentum* MTCC 8711  *Lactobacillus oris* | EQC58604.1  AMS08876.1 | 99  97 |
| EH277_00435 (249) | Polymerization and chain-length determination | polysaccharide biosynthesis tyrosine autokinase | *Lactobacillus fermentum* | WP_049184269.1 | 95 |
| EH277_00440 (256) | Polymerization and chain-length determination | possible protein-tyrosine kinase  Tyrosine-protein kinase EpsD | *Lactobacillus fermentum* TDS030603  *Lactobacillus reuteri* | BAI67352.1  CUR37483 | 97  67 |
| EH277_00445 (313) | UDP-sugars biosynthesis | NAD-dependent epimerase/dehydratase family protein | *Lactobacillus fermentum* | WP_104877737 | 99 |
| EH277_00450 (229) | Synthesis of the repeating unit | priming glycosyltransferase | *Lactobacillus fermentum* | CDN26049.1 | 97 |
| EH277_00455(277) | Synthesis of the repeating unit | Glycosyl transferase family 2 | *Lactobacillus mucosae* | SEK17814.1 | 57 |
| EH277_00460 (292) | Synthesis of the repeating unit | glycosyl transferase | *Lactobacillus plantarum* | WP_120783750.1 | 37 |
| EH277_00465 (405) | Export | putative membrane protein  epsG family protein | *Lactobacillus reuteri* ATCC 55730  *Clostridium baratii* | ABS84226.1  AIY83234 | 34  26 |
| EH277_00470 (380) | Export | CDP-glycerol-glycerophosphate glycerolphosphotransferase | *Lactobacillus salivarius* | WP_081537115.1 | 41 |
| EH277_00475 (300) | Synthesis of the repeating unit | putative capsular polysaccharide synthesis protein | *Lactobacillus reuteri*  *Lactobacillus brevis* KB290 | WP_078009514.1  BAN07212 | 37  31 |
| EH277_00480 (131) | Others | glycerol-3-phosphate cytidylyltransferase | *Bifidobacterium thermacidophilum* | WP_033506799.1 | 86 |
| EH277_00485 (348) | Synthesis of the repeating unit | glycosyltransferase family 2 protein  putative glycosyltransferase EpsJ | *Lactobacillus agilis*  *Acetobacterium wieringae* | WP_101811490.1  OFV71345.1 | 32  34 |
| EH277_00490 (320) | Synthesis of the repeating unit | glycosyltransferase family 2 protein | *Lactobacillus fermentum* | WP_114684435.1 | 96 |
| EH277_00495 (510) | unknow | hypothetical protein | *Lactobacillus fermentum* | WP_128492753.1 | 99 |
| EH277_00500 (186) | unknow | hypothetical protein | *Lactobacillus amylovorus* GRL 1112  *Lactobacillus casei* | ADQ58760  WP_016364976.1 | 54  34 |
| EH277_00505 (263) | unknow | hypothetical protein (DUF1828 domain-containing protein) | *Lactobacillus reuteri* | KGE70608.1 | 53 |
| EH277_00510 (118) | Transposase | transposase | *Lactobacillus fermentum* 3872 | N573_000575 | 85 |
